# Looking to the Future: A Synthesis of New Developments and Challenges in Suicide Research and Prevention

**DOI:** 10.3389/fpsyg.2018.02139

**Published:** 2018-11-27

**Authors:** Rory C. O’Connor, Gwendolyn Portzky

**Affiliations:** ^1^Suicidal Behaviour Research Laboratory, Institute of Health & Wellbeing, University of Glasgow, Glasgow, United Kingdom; ^2^Unit for Suicide Research, Flemish Centre of Expertise in Suicide Prevention, Ghent University, Ghent, Belgium

**Keywords:** suicide, theory, challenges, clinical, risk factors, new technologies

## Abstract

Suicide and attempted suicide are major public health concerns. In recent decades, there have been many welcome developments in understanding and preventing suicide, as well as good progress in intervening with those who have attempted suicide. Despite these developments, though, considerable challenges remain. In this article, we explore both the recent developments and the challenges ahead for the field of suicide research and prevention. To do so, we consulted 32 experts from 12 countries spanning four continents who had contributed to the *International Handbook of Suicide Prevention* (2nd edition). All contributors nominated, in their view, (i) the top 3 most exciting new developments in suicide research and prevention in recent years, and (ii) the top 3 challenges. We have synthesized their suggestions into new developments and challenges in research and practice, giving due attention to implications for psychosocial interventions. This Perspective article is not a review of the literature, although we did draw from the suicide research literature to obtain evidence to elucidate the responses from the contributors. Key new developments and challenges include: employing novel techniques to improve the prediction of suicidal behavior; testing and applying theoretical models of suicidal behavior; harnessing new technologies to monitor and intervene in suicide risk; expanding suicide prevention activities to low and middle-income countries; moving toward a more refined understanding of sub-groups of people at risk and developing tailored interventions. We also discuss the importance of multidisciplinary working and the challenges of implementing interventions in practice.

## Introduction

Suicide and attempted suicide are major public health concerns. At least 804,000 people take their own lives annually and 25 times that number attempt suicide (WHO, 2014). In recent decades, there have been many welcome developments in understanding and preventing suicide, as well as good progress in intervening with those who have attempted suicide. Despite these developments, though, many challenges remain. In this article, we explore both the recent developments and the challenges ahead for the field of suicide research and prevention. Instead of relying solely on our individual perspectives, we consulted experts in suicide research and prevention from across the globe. To this end, we contacted all of the contributors to the 2nd edition of the *International Handbook of Suicide Prevention* (O’Connor and Pirkis, 2016) and asked them to nominate, in their view (i) the top 3 most exciting new developments in suicide research and prevention in recent years, and (ii) the top 3 challenges in the field of suicide research and prevention. We were fortunate to receive responses from about one third of the authors representing 12 countries and spanning four continents (see section “Acknowledgments”). We reviewed their nominations, combined them where they described an overarching theme and then classified them into whether they referred to research or practice^1^. We also expanded upon their brief comments and added supporting references (largely in the new developments sections) to elucidate the specific development or challenge. Needless to say, these are fuzzy boundaries and some of the entries could be classified into more than one category. It is important to highlight that this Perspective article is not a review of the literature, although we did draw from the suicide research literature to obtain evidence to elucidate the responses from the contributors. Given the nature of the task, some of the new developments/challenges are very specific and others are more general. The interpretations of the contributors’ submissions are ours and do not necessarily reflect those of the individual contributors. It is also important to emphasize that this appraisal of the developments and challenges within the field is not exhaustive and it reflects our biases and those of the contributors; it is our combined view (together with our international experts’ views) of the recent past within the field and our thoughts about the future. It could also be argued that, as the contributors all wrote chapters for a single handbook, they are all like-minded individuals with a particular view on suicide prevention. Nonetheless, we believe that this synthesis will be helpful to guide those involved in suicide research and prevention as it highlights hot topics in the field. We also highlight at the outset that despite the developments in understanding suicide risk, our ability to predict suicide remains no better than chance and in many countries across the globe suicide rates continue to increase (O’Connor and Pirkis, 2016; Franklin et al., 2017).

### New Developments in Research

The use of new technologies (including social media and naturalistic real-time monitoring via smartphones) to increase understanding of suicidal behavior and to better identify suicide risk were the most frequently cited new research developments nominated by our contributors (see Panel 1). With the proliferation of smartphone ownership globally, in low- and middle-income countries (James, 2014) as well as in high-income countries, the growth in interest is not surprising (de Beurs et al., 2015). Given the field’s continued inability to predict suicidal behavior with sufficient sensitivity/specificity (O’Connor and Nock, 2014; de Beurs et al., 2015; Franklin et al., 2017), the use of smartphone technologies affords the opportunity to assess risk factors repeatedly, in real-time and in naturalistic settings (de Beurs et al., 2015; Michaels et al., 2015). It is hoped that the use of such technologies will better capture the ‘waxing and waning’ nature of suicidal ideation (Joiner and Rudd, 2000; Zisook et al., 2009) and account for the complex interaction between the risk factors which predict the transition to suicide attempts (de Beurs et al., 2015; O’Connor and Kirtley, 2018). If the promise of new technologies is realized, individuals or clinicians may be able to better identify windows of acute risk in real-time (based, in part, on social media and moment-to-moment monitoring), alert others and hopefully receive interventions to alleviate that risk. Needless to say, there are many practical and ethical barriers that have yet to be overcome, but they are not insurmountable.

**Panel 1:** New developments in Research.

1. Use of new technologies and social media (such as a naturalistic real-time monitoring) to increase understanding of suicidal behavior and to identify those at heightened suicide risk. 2. Development of new theories of suicidal behavior which seek to understand specific factors and processes involved in suicidality. 3. The implications of the NIMH’s Research Domain Criteria Framework for suicide research. 4. Development of implicit association tasks, given their novelty and implications for theory and potentially treatment development. 5. Big data and machine learning approaches to identify novel risk factors. 6. Development of a novel procedure for examining proximal risk factors for suicidal behavior using retrospective timeline follow back methodologies. 7. The application of network analysis to understanding suicide risk. 8. New developments in brain imaging and epigenetics. 9. Greater appreciation of the interdisciplinary understandings of suicidality including understanding social factors, social disconnection, social roles and social disadvantage. 10. Recognition of the importance of postvention and those with lived experience as key to suicide research and prevention activities.

The use of ecological momentary assessment (via mobile phones) has already been shown to be feasible (Palmier-Claus et al., 2011; Husky et al., 2014) and it offers considerable promise in enhancing our prediction of the suicidal ideation–suicide attempts gap (Myin-Germeys et al., 2009). In terms of social media, Twitter and Facebook are now being harnessed to help understand the transmission of risk of suicide and self-harm. For example, a recent study from Japan has shown that social media coverage of celebrity suicides varies as a function of characteristics of the celebrity, with large volumes of traffic happening when the celebrity is a relatively young entertainer (Ueda et al., 2017). Also in Asia, text mining and machine learning approaches have been applied to Chinese social media to identify language markers of suicide risk and emotional distress (Cheng et al., 2017). Social media is also being used a lot by young people as a means of communicating distress (Marchant et al., 2017). More generally, although Facebook is rolling out safety protocols that aim to identify social media users at-risk of suicide via their online posts, it is not clear whether such interventions are effective. As such initiatives develop it is vitally important that they are rigorously evaluated and potential unintended consequences (e.g., do they lead to more social isolation as these initiatives lead to a reduction in sharing on social media?) considered. These developments have important implications for theories of suicide risk and contagion as well as suicide prevention efforts more generally. As noted above, although these developments are exciting, best practice guidelines need to be developed to ensure these technologies are implemented safely and ethically (Michaels et al., 2015).

The second most frequently cited development was the growth in theories of suicidal ideation and behavior. This is, perhaps, unsurprising given that at least 12 theories have been put forward since the mid-1980s (O’Connor et al., 2016) beginning with Shneidman’s cubic model of suicide (Shneidman, 1985) [obviously Durkheim pre-dates all of these contemporary models (Durkheim, 1897)]. Three of the recent predominant theories (the interpersonal theory, the integrated motivational-volitional model and the three step theory) have received considerable research attention; each fitting within the ideation–to–action framework (Joiner, 2007; Van Orden et al., 2010; O’Connor, 2011; Klonsky and May, 2015; O’Connor et al., 2016; O’Connor and Kirtley, 2018; O’Connor and Portzky, 2018) which describes those theories which posit that the factors associated with suicidal ideation are distinct from those that govern behavioral enaction, i.e., a suicide attempt/suicide (O’Connor and Nock, 2014; Klonsky et al., 2016, 2017).

Although each of these theories emphasizes different factors that lead to the emergence of suicidal ideation and behavior, they have shaped our understanding of the suicidal process: historically, theories of suicide did not explicitly specify the conditions that led to suicidal ideation as being distinct from those associated with a suicide attempt/death by suicide. In brief, these new theoretical developments have been important not only to enhance understanding of the complexities of the suicidal process but they are also forming the basis for the development of psychological interventions to reduce risk of suicide and self-harm. For example, a recent brief psychosocial intervention (a volitional helpsheet) which draws from the integrated motivational–volitional model of suicidal behavior offers promise in reducing risk of repeat self-harm in some individuals following a suicide attempt (Armitage et al., 2016; O’Connor et al., 2017). The recent focus on safety planning and crisis response planning interventions is also consistent with the ideation-to-action framework (Stanley and Brown, 2012; Bryan et al., 2018) and are welcome additions to the field.

Given that suicide and suicide attempts are transdiagnostic phenomena, the move away from a focus on individual mental disorders coupled with the introduction of the National Institute of Mental Health’s Research Domain Criteria (RDoC) was identified as a positive development for the field (Glenn C.R. et al., 2017). Indeed, in a novel approach Glenn and colleagues conducted a meta-analysis of transdiagnostic dimensions (Glenn et al., 2018). Rather than focusing on risk factor domains, they viewed the predictors of suicidal behavior through the lens of the RDoC domains. Perhaps unsurprisingly, they found that limited prospective research, to date, fits within the RDoC transdiagnostic framework and even less addresses protective factors. Where there was evidence, it tended to be for the Negative Valence Systems domain (e.g., hopelessness) but there was also growing evidence for suicide theory-related factors (e.g., burdensomeness, defeat/entrapment) (Van Orden et al., 2010; O’Connor, 2011; Glenn et al., 2018). One of the key messages for future research from Glenn and colleagues’ recent meta-analysis is that we need to move beyond “the ‘usual suspects’ of suicide risk factors (e.g., mental disorders, sociodemographics) to understand the processes that combine to lead to this deadly outcome.”(Glenn et al., 2018).

The use of innovative study designs and new techniques were also identified as important developments. Four such designs or techniques were highlighted by our contributors. The first is the use of big data and machine learning. Consistent with other areas of psychopathology, the statistical and computing power of big data and machine learning is now being applied to suicide risk assessment. Such approaches have the advantage of being able to combine a large number of risk factors in the prediction of suicide risk and they have already been shown to be moderately successful (Franklin et al., 2017; Hettige et al., 2017; Kessler et al., 2017). As the machine learning field develops, it will be interesting to determine the extent to which the algorithms can be applied to real-world clinical contexts to inform treatment planning (see also Research challenges below).

The second technique is the retrospective timeline followback (TLFB) methodology (Sobell and Sobell, 1992) which systematically assesses behaviors/events in the days/weeks preceding an index event. Although TLFB is not new (Sobell and Sobell, 1992), its application within a case-crossover design to understand suicide risk in the days and hours preceding a suicide attempt is novel. Building on the work of Conner et al. (2012) showing that interpersonal stressful life events may lead to a suicide attempt within the same day, Bagge et al. (2013) conducted a TLFB study but focused on the 48 h preceding a suicide attempt. In the first study of its kind, they demonstrated that negative life events (NLEs) were triggers for a suicide attempt and that NLEs occurred more often on the day of, rather than the day before, a suicide attempt (Bagge et al., 2013). Given that we know relatively little about the factors that trigger suicide attempts in the preceding hours, we would urge others to consider employing the TLFB method.

Third, the innovative work on predicting suicidal behavior using implicit cognitions toward death has been a welcome addition to the literature (Nock et al., 2010). Implicit cognitions assess one’s automatic associations with life or death. For example, it may be possible that an individual’s unconscious association with wanting to live or die changes as their mood decreases – and this could be incorporated into a real-time warning system. Not only do implicit measures overcome the issue of one’s reluctance to disclose suicidal intent but they also tap directly into the automatic processes that govern behavior (alongside reflective processes) (Strack and Deutsch, 2004). Although this is an exciting development, there are many unanswered questions, including, how stable are implicit cognitions, how are they formed, over what time frame and with whom are they predictive as well as how are they related to existing risk factors? (Dickstein et al., 2015; Hussey et al., 2016; Glenn J.J. et al., 2017). Indeed, a recent study, conducted across two research labs in United States and Scotland, found that implicit attitudes can be activated by low mood in those with a suicidal history (Cha et al., 2018).

Finally, network analysis is a new statistical technique that has been applied to psychopathology in general and suicidal behavior specifically in recent years (de Beurs, 2017; de Beurs et al., 2017; Fried et al., 2017). The advantage of network analysis is that it allows researchers to investigate the complex associations between risk factors or symptoms. It also determines which symptoms are central within a network thereby highlighting specific treatment targets with the potential to be most powerful in reducing risk of suicidal behavior. To our knowledge only one prospective study of suicidal behavior has been published to date (de Beurs et al., 2017) so it is unclear which symptoms will have optimal predictive power. Nonetheless, we urge researchers to embrace this new statistical technique.

The past 20 years of research has also been witness to new developments in brain imaging techniques and epigenetics (van Heeringen, 2014; van Heeringen and Mann, 2014; Sudol and Oquendo, 2016). With respect to the former, in a recent review of 12 neuroimaging techniques, 5 yielded important findings specific to suicide attempts [namely, magnetic resonance imaging (MRI), diffusion tensor imaging (DTI), functional MRI (fMRI), positron emission tomography (PET), and single-photon emission tomography (SPECT)] (Sudol and Oquendo, 2016). Taken together the brain imaging studies have identified both structural and functional abnormalities in the prefronal and limbic areas of the brain in individuals with a suicidal history. Obviously, brain imaging is only part of the answer in piecing together the suicide risk puzzle, but such approaches continue to make an important contribution to our understanding of suicide-specific markers of risk. For example, such research helps to explain, in part, the deficits in emotional regulation and decision-making that often characterize suicide risk (Sudol and Oquendo, 2016).

The neurobiology of suicidal behaviour and epigenetics were also highlighted. There is an established body of research, employing different study designs (including *in vivo*, experimental and post-mortem techniques) finding that impairments in the serotonergic and hypothalamic-pituitary-adrenal axis stress response systems, in particular, are associated with increased vulnerability to suicide (van Heeringen and Mann, 2014; Lutz et al., 2017). The growing recognition of the influence of external factors, including early life adversity, on gene expression, has also led to a step change in our interpretation of the relationship between stress, mental disorders and suicide vulnerability (van Heeringen and Mann, 2014; Lutz et al., 2017). Indeed, there may be unique epigenetic processes (including altered cortisol responses and altered glutamate signaling) at play that increase suicide risk, with Lutz et al. (2017) arguing that understanding the former “has contributed to one of the most meaningful changes to the neuroscience landscape in the past 15 years (Lutz et al., 2017)”. There is also evidence that microRNAs may play a critical role in suicide risk (Serafini et al., 2014). Finally, it is also noteworthy that neurobiological scientists are incorporating key psychological and social factors into their modeling of suicide risk (Turecki and Brent, 2016; Lutz et al., 2017).

No single discipline can address the complex challenge of understanding risk, as suicide is the end product of a complex interplay of neurobiological, psychological, and social processes. Indeed, social factors, including social isolation, disconnection (Stack, 2000; Macrynikola et al., 2018), loneliness (Bennardi et al., 2017) and social disadvantage (Batty et al., 2018) were flagged by a number of respondents as key determinants of suicide risk which have received welcome attention in recent years. What is more, social disconnection and social isolation (O’Connor and Nock, 2014) (including the absence of social support) feature in the interpersonal theory of suicide (Van Orden et al., 2010) the integrated motivational–volitional model of suicidal behavior in particular (O’Connor and Kirtley, 2018) and the 3 step theory (Klonsky and May, 2015).

The social context is crucial to understanding suicide risk especially given the evidence that suicide is socially patterned being significantly more prevalent in areas of social disadvantage compared to more affluent areas (Platt, 2016; Batty et al., 2018). Although there have been some developments in understanding how changes in the social role may contribute to suicide risk, more needs to be done to better understand how conceptualizations of masculinities may elevate suicide vulnerability (Scourfield et al., 2012). Finally, an incredibly positive development in the field in recent decades has been the recognition of the importance of postvention and those with lived experience (including suicide attempt survivors and suicide bereavement survivors) as key to suicide research and prevention activities. Lezine recently described the vital work of suicide prevention through personal experience (Lezine, 2016) which we would urge everyone involved in suicide research to read.

### Challenges in Research

The most frequently cited challenge was the issue of the reliability of suicide data due to low base rates and small samples in intervention studies, in particular (see Panel 2). This is a significant issue as we endeavor to build the evidence base for what works to prevent suicide. The lack of evidence for the efficacy of psychological and pharmacological treatments to prevent suicide specifically may be, in part, attributable to this issue of scale. As suicide is a low base-rate outcome, almost all clinical RCTs have been underpowered to detect changes in suicide rates and, at best, they have employed suicide attempts as the primary outcome. Even then, the sample sizes have tended to be modest, thereby precluding the a priori investigation of whether interventions may be effective in some groups of participants but not in others. For example, even where there is evidence for clinical efficacy of psychosocial interventions to reduce self-harm (Hawton et al., 2016), we cannot say whether they are effective for men as well as for women. One potential solution might be to include suicide-related measures as secondary outcomes in all psychological treatment trials and then potentially aggregating findings across studies, where appropriate (Holmes et al., 2018). Another solution relates to the second challenge. Given that the size of suicide research field is relatively modest and the challenge around statistical power, the establishment of national and international networks may facilitate large-scale research opportunities.

**Panel 2:** Challenges in Research.

1. Problem with reliability of suicide data due to low base rates and small samples in intervention studies. 2. More investigation of suicide deaths. Recent research has also disproportionately focused on suicide ideation and attempts as outcome variables. 3. Establishment of national and international networks within the research community to enable large-scale evaluation of prevention activities, including multicentre trials. 4. Scarcity of translational research in terms of research evidence informing policy (e.g., means restriction, government austerity measures) and practice (availability of treatments). 5. Need for novel risk factors. Prediction has not been improved since the 1960s, and is restricted by the use of methods that are unlikely to provide clinically useful markers of risk of suicidal behavior. 6. Limited research on short-term/acute risk [most prospective prediction studies are conducted over long periods of time (years), which have limited clinical utility]. 7. We need to develop protocols to include high-risk individuals in suicide research as now they are frequently excluded. 8. Interdisciplinary research. Continuing to increase linkages with other disciplines and areas of research and policy (especially interdisciplinary work needed due to new technological developments). 9. More attention needed on social context/social factors in suicide risk including disadvantage and social media. Delivery of more research in low and middle income countries. 10. Concerns about “big data” (e.g., to create real time risk monitoring algorithms does not adequately appreciate the person-specific nature of suicide warning signs). 11. More research/knowledge regarding the transitions from ideation to attempt is required. 12. More research/knowledge regarding factors that differentiate those who make low-lethality attempts versus those who make high-lethality attempts. 13. Understanding the causes of suicidal behavior in persons who do not have mental illnesses. 14. Tensions in research focus: ensure that all areas of research (biology, psychology, epidemiology, and social context) receive appropriate attention.

Relatedly, the scarcity of translational research was also noted as a challenge. This is an important consideration, as new developments in suicide research are frequently not translated into saving people’s lives. The issue of translation relates to ensuring that research evidence, for example the potential effect of government austerity policies on health, is translated into a change in government policy. Another example would be the limited interest in some countries in implementing strategies in restricting the access to the means of suicide (see also practice challenges). We also need to re-double efforts to increase the likelihood of evidence-based treatments for suicidal behavior being accessible to those who need them. Consistent with the call within the *Lancet Psychiatry* Commission on psychological treatments research (Holmes et al., 2018) to focus on implementation, the same onus is on suicide researchers specifically. The second challenge is also related to the issue of scale, arguably. Excluding large-scale national linkage database type research, the majority of risk factors/predictors research in the field has tended to focus on suicide ideation and attempts as outcome variables – as well as being limited to a small number of predictors. This is an important limitation and perhaps the establishment of the networks or suicide research hubs (as suggested above) can address this dearth in the literature.

As noted earlier in this paper, the field of suicide research has to continue to move beyond traditional risk factors (e.g., psychiatric illness) and to embrace complexity. We need a renewed focus on novel risk factors and multivariable risk factors; this is especially urgent as our ability to predict suicide has not improved since the 1960s and it remains no better than chance (Franklin et al., 2017). With a few exceptions, limited research has focused on short-term acute risk, with researchers often directing their attention to the long-term follow-up of patients who are high risk (O’Connor et al., 2009; Glenn et al., 2016; Glenn J.J. et al., 2017). Although the latter is important, arguably such studies have limited clinical utility and given that there is increased risk of suicidal behavior in the days and weeks following discharge from hospital (Owens et al., 2002; Chan et al., 2016; Ribeiro et al., 2016), we need to know more about this acute window. In addition, next on the list of challenges relates to the systematic exclusion of high-risk participants from our studies. We need to develop new protocols to include the very people who are most likely to benefit from our research findings. Obviously there are ethical and safety challenges, but, arguably it is unethical to exclude this vital group of participants in research studies.

There have been considerable improvements in terms of interdisciplinary working in recent years. For example, in 2008, one of us (RCOC) organized the European symposium on suicide and suicidal behavior (ESSSB12; the leading suicide research conference in Europe) in Glasgow, Scotland. This was a huge success; it had the theme of ‘working together to prevent suicide: research, policy and practice’ and there were countless examples of interdisciplinary research showcased therein. What is more, 10 years later, the other one of us (GP) organized the same conference in Ghent, Belgium (ESSSB17), again with the explicit plea for high-quality *multidisciplinary* research. Despite these developments, we should not be complacent; there remains a considerable need to continue building the linkages across disciplines and to involve everyone who has a stake in suicide prevention in our research. Indeed one of our experts highlighted that such working is essential especially to maximize the opportunities afforded by new technological developments. The lack of an international consensus in the terminology used in the field remains a challenge (Silverman, 2016); mutual cross-disciplinary respect is also required to ensure that interdisciplinarity flourishes.

Although Durkheim, one of the pioneers in suicide research, highlighted the central role of social context and social factors in the etiology of suicide in 1897 (Durkheim, 1897), without question, their roles have not received adequate attention in the 121 years since. This is especially alarming given the scale of the socio-economic gradient in suicide and the established relationships with unemployment and disadvantage (Platt, 2016; Batty et al., 2018). However, the renewed focus on adverse childhood experiences in suicide risk is welcome (Turecki and Brent, 2016; O’Connor et al., 2018). At the other end of the social context spectrum are social media influences and other volitional factors (O’Connor and Kirtley, 2018) which are implicated in suicidal behavior and self-harm. We do not know enough about how such factors act to increase suicide risk in terms facilitating social modeling and increasing cognitive accessibility (O’Connor and Nock, 2014; O’Connor et al., 2014; Mars et al., 2015; Biddle et al., 2016). Another major challenge is the relative dearth of research into suicide in low and middle-income countries (LMIC). Despite the fact that the vast majority of the world’s suicides occur in LMIC (WHO, 2014), (although this is changing) there is still insufficient research focus in LMIC.

The advent of machine learning techniques and the use of “big data” algorithms were identified as exciting new developments in the preceding section. However, caution is also urged by our contributors as such algorithms may not adequately appreciate the person-specific nature of suicide warning signs. This highlights the importance of adopting a multi-method approach to understanding suicide risk. As noted above, no single approach, method or discipline has all of the answers; if we are to make further progress in predicting suicide, this is most likely to succeed if we integrate multiple approaches and crucially we should not throw the baby out with the bathwater.

The next three challenges are inter-related as they each refer to a more fine-grained appreciation of sub-types of individuals who are at increased/decreased risk. The first in this triad is also featured in the new developments section, as it calls for a better understanding of the transition from suicidal thoughts to suicide attempts/suicide. As noted above, the ideation to action theories (Van Orden et al., 2010; Klonsky and May, 2015; O’Connor and Kirtley, 2018) are beginning to address this important challenge more systematically, though the findings from the World Mental Health surveys have also contributed to our understanding of these pathways (Nock et al., 2008). Nonetheless, we believe that this is one of the biggest challenges in the field as there are so many gaps in our understanding of this transition (Nock et al., 2008; O’Connor et al., 2012; Dhingra et al., 2015). We also need more research regarding the factors that differentiate between those who make low-lethality attempts versus those who make high-lethality attempts. Although a few high quality studies exist (Rivlin et al., 2013; Marzano et al., 2016; Anestis et al., 2018), focused on specific populations, more in-depth research is required. In addition, although there has been growing focus on the relationship between sleep and suicide risk (Littlewood et al., 2017), among those who have attempted suicide, insomnia may be associated with a more violent method (Pompili et al., 2013).

The final challenge in this group calls for increased understanding of the causes of suicidal behavior in people who do not have psychiatric illnesses. Despite the evidence that most people in Western countries who die by suicide have a diagnosed mental illness (Cavanagh et al., 2003), there are cultural variations (Phillips, 2010) and some argue that the association between mental illness and suicide is not as marked as is commonly reported (Hjelmeland and Knizek, 2017). As the relationship between mental illness and suicide is often ascertained via a psychological autopsy, researchers should implement the recommendations on the next generation of psychological autopsy studies that aim to increase the accuracy of data collected (Conner et al., 2011). These latter three challenges highlight the more general point that we still often treat people at risk of suicide as an homogeneous group but we need to move beyond such characterization and identify distinct profiles of people at risk: the precision medicine approach.

As the determinants of suicide are many, spanning neurobiology, psychology and social factors, it is not surprising that there is a tension between where our research effort should be focused. A number of contributors argued for an increase in attention to the neurobiological determinants of suicide as these would inform treatment targets and a reduced focus on epidemiology. As noted above, no one discipline has all of the answers so it is important that all areas receive attention.

### New Developments in Practice

The most frequently reported new development in research (i.e., the use of new technologies) is also the most frequently mentioned new development in practice (see Panel 3). So called ‘new’ technologies such as the internet and mobile phones not only increase our understanding of suicidal behavior but they can also be harnessed for treating and connecting with individuals who are suicidal (Hom et al., 2015; de Beurs et al., 2015; Hetrick et al., 2017; Nuij et al., 2018).

**Panel 3:** New developments in Practice.

1. The use of new technologies, the internet and mobile phones for the treatment of (and connecting with) suicidal patients, vulnerable young people, older adults at risk and those who are not in contact with clinical services. 2. A growth in suicide prevention interventions including psychosocial interventions to reduce suicidal behavior that have been evaluated in RCTs and thus having demonstrated efficacy. 3. The growth of clinical trials on suicide-specific interventions, almost all of which acknowledge that suicide must be the focus of treatment rather than viewing it as a symptom of some other mental disorder. 4. The identification of very high-risk groups that could benefit specific interventions. 5. Greater involvement of and attention to the insights of those with lived experience of suicide in the design and improvement of interventions and services for suicide prevention. 6. Growing evidence on effectiveness of school-based programs. 7. New anti-psychotic drug treatments.

Given that less than one third of suicidal individuals seek help or make use of mental health services it is clear that there are numerous barriers to traditional forms of treatment such as stigma and shame, low perceived need, a preference for self-management, availability and high cost of care (Bruffaerts et al., 2011; Andrade et al., 2014). Online interventions are well placed, therefore, to overcome many of these barriers as they are easily accessible anywhere at anytime, at low cost, and are mostly anonymous or highly confidential (Hom et al., 2015).

Although there have been several studies sharing positive effects of online interventions in the reduction of suicidal ideation (Christensen et al., 2013; Saulsberry et al., 2013; Williams and Andrews, 2013; Mewton and Andrews, 2015) many of these online interventions were developed to manage depression and were not designed to target suicidal ideation specifically. In a rare exception, van Spijker et al. (2014) examined the effectiveness of an online intervention specifically targeting suicidal ideation in a Dutch community sample. The results were promising, showing a significant reduction in suicidal ideation thereby highlighting the potential for managing suicidal ideation via an online intervention. However, in this study individuals with severe suicidal ideation were excluded and a controlled follow-up period was missing. This online intervention has also been examined in an Australian community sample by use of a randomized controlled trial (van Spijker et al., 2018). Although the intention-to-treat analyses showed significant reductions in the severity of suicidal thinking at post-intervention, 6 and 12 months, no overall group differences were found. It is, thus, clear that more research is needed and it is encouraging that this online intervention is currently being examined in other countries such as Denmark and Belgium (De Jaegere, submitted).

Although it is not surprising that suicide experts have focused on suicide-specific tailored interventions, it is important to direct attention at suicidal thoughts and behavior as treatment targets rather than viewing them exclusively as symptoms or epiphenomena of mental disorder. Indeed, many previous studies have focused on the treatment of depression and viewed suicidal ideation as a secondary outcome that may improve if the intervention for depression is effective. Therefore, the growth in clinical trials that have investigated the efficacy of suicide-specific prevention and intervention strategies aimed at reducing suicidal behavior is a welcome development.

To this end, there has been growing evidence for suicide-specific *psychosocial* interventions which show promise in reducing suicidal behavior (Hawton et al., 2016). For example, Gysin-Maillart et al.’s (2016) 24-month follow up randomized clinical trial (the Attempted Suicide Short Intervention Program; ASSIP) of a novel brief therapy for patients who had attempted suicide was effective in reducing suicidal behavior in a real-world clinical setting. ASSIP was associated with a circa 80% reduced risk of repetition of at least one suicide attempt and ASSIP participants spent 72% fewer days in the hospital during follow-up compared to controls. ASSIP consists of three therapy sessions followed by regular contact through personalized letters over 24 months (Tarrier et al., 2008). The development of the Collaborative Assessment and Management of Suicidality (CAMS) approach has been an exciting new development in the field which also offers considerable promise (Comtois et al., 2011; Jobes, 2016).

Another brief psychological intervention with a volitional helpsheet (VHS) has also yielded promising results (O’Connor et al., 2017). Although the VHS had no overall effect, *post hoc* analyses suggested that this brief adjunct intervention might be effective in reducing the number of self-harm repetitions following a suicide attempt among those with a self-harm history. In another modest sized RCT which compared a 6-week telephone-based positive psychology (PP) intervention with a cognition-focused (CF) control intervention, those who received the CF intervention reported greater improvements in hopelessness at 6 weeks but not at 12 weeks (Celano et al., 2017). This study recruited patients who had recently been hospitalized for depression and suicidal ideation or behavior. There was also greater improvement in suicidal ideation, depression and optimism at 6 and 12 weeks after receipt of the CF intervention. Contrary to the authors’ hypothesis, however, it was the CF intervention that was superior in improving hopelessness, other suicide risk factors and positive psychological constructs rather than the PP intervention (Celano et al., 2017).

These latter studies add to the established evidence that CBT has a significant effect in reducing suicidal behavior (O’Connor and Nock, 2014; Hawton et al., 2016). However, a systematic review and meta-analysis from 10 years ago still nicely summarizes the gaps in our knowledge (Tarrier et al., 2008). Tarrier et al.’s (2008) subgroup analyses confirmed significant treatment effects for CBT in adult samples (but not in adolescent samples), for individual treatment delivery (but not for group administration) and for CBT when compared to minimal treatment or treatment as usual (but not when compared to another active treatment; Tarrier et al., 2008).

As noted in the new research developments section, the insights offered by those with lived experience into the design and improvement of suicide prevention programs were also highlighted by our experts. Although their contributions have taken different forms, there is now a considerable body of qualitative evidence on the aftermath of a suicidal crisis or attempt based on interviews with those with lived experience. Such studies have deepened our understanding of what it is like to be suicidal and how to help those who are vulnerable (Lin et al., 2009; Oliffe et al., 2012; Vatne and Naden, 2016).

More recently, attention has been paid to ensuring that such insights and testimonials inform the development and implementation of suicide prevention programs. For example, Jones et al. (2018) explored the views of health and human service workers with regard to the development of a suicide prevention training program. This included meaningful involvement of someone with lived experience in the development and delivery of the training. The authors concluded that the inclusion of a person with lived experience of suicidality resonated strongly with the participants and provided a powerful learning experience for those involved. A strong positive element was that the person with lived experience gave participants crucial insights into how to have conversations around suicide and how best to ask the questions about thoughts of suicide directly. Although this study indicates the positive effect of including people with lived experience the authors also caution that the inclusion of a person with lived experience must be appropriate and safe. In addition, despite the progress in involving people with lived experience to improve research and practice, we should not be complacent; we have a long way to go in terms of maximizing their involvement to the mutual benefit of all.

The growth in specific interventions for high-risk groups was also highlighted as an important new development. By way of an example, Vijayakumar et al. (2017) reported the effectiveness of CASP, an intervention by community volunteers among refugees, to reduce suicidal behavior. This intervention involves contact between community volunteers and refugees and the use of safety planning cards. The findings from the RCT were positive as the intervention was associated with a significant reduction in suicidal behavior among the refugees. Interventions for another high-risk group, older adults, are also encouraging. Specifically, a recent systematic review of interventions to prevent and reduce suicidal behavior in older people showed that several interventions are effective, with at least some evidence for multi-faceted primary care-based depression screening and management programs, pharmacotherapy and psychotherapy, and telephone counseling (Okolie et al., 2017). In short, there is the increasing recognition that the one size fits all approach to suicide prevention initiatives is not effective and we must tailor interventions to fit specific at-risk populations.

The growing evidence for effective school-based programs was also highlighted by our experts. There is now some evidence for peer-support prevention programs and skills-based training programs, which show positive outcomes regarding coping skills and referral to help (Katz et al., 2013; Hetrick et al., 2014, 2017). There is also some evidence that screening programs have some utility in identifying young people at risk (Robinson et al., 2013). Although it is important to note that there are many challenges with screening programs including the issue of false positives and the need for available resources to support those who are identified as high risk. School-based awareness programs have also been shown to significantly improve knowledge, attitudes and help-seeking behavior (Cusimano and Sameem, 2011). However, the most promising findings are those from the Saving and Empowering Young Lives in Europe (SEYLE) study (Wasserman et al., 2015). In this multicentre cluster-randomized controlled trial, adolescents who received the youth aware of mental health (YAM) program reported a significant reduction in incident suicide attempts and severe suicidal ideation compared with the control group at 12-month follow-up. As with all areas of practice, it is important to replicate these findings.

New anti-psychotic drug treatments were identified as a final new development. Although there is “at least modest evidence suggesting that antipsychotic medications protect against suicidal risk” (Kasckow et al., 2011), a combination of psychosocial and pharmacological management is recommended. Of all the anti-psychotic medications, though, the best evidence is for clozapine (Kasckow et al., 2011). Consistent with the pharmacological treatment literature more generally, there remains a dearth of large-scale treatments of anti-psychotic medications.

### Challenges in Practice

The experts’ opinions regarding the challenges in practice were diverse, with the majority of the suggestions only being mentioned by one expert (see Panel 4).

**Panel 4:** Challenges in Practice.

1. More research and robust evidence are needed for universal and selective prevention strategies. 2. Need to change attitudes, beliefs and knowledge regarding the preventability of suicide in general and the utility of means restriction and means safety in particular. 3. Ensuring that those in contact with health and social care services receive high a quality chain of care. The development of better linkages between crisis intervention, statutory services, therapists and the wide range of community and voluntary sector organizations involved in suicide prevention is vital. 4. A need for more evidence to inform and guide best practice for the evaluation, treatment, and follow-up of people who present to hospitals with self-harm. 5. Better knowledge about how to help men at risk of suicide, particularly how to motivate prevention efforts and treatment engagement. 6. Identify and treat first onset suicidal ideation in adolescents. 7. Learn from other areas of public health including studying the effects of a dental health approach to improve mental health and prevent suicide. 8. Much more attention to the lived experience of people in the development of interventions and service design. 9. Very little suicide-specific treatment is available in the real world. There is a need to improve training and dissemination of evidence-based approaches. 10. Integration of clinical knowledge into the evidence base.

The only challenge which was mentioned multiple times was the need for more research and evidence for universal and selective prevention strategies. This is, perhaps, unsurprising as the USI (Universal, Selective, Indicated) prevention model forms the basis for much suicide prevention activity worldwide. Despite its use as an overarching framework, it is obvious from the research literature that there are extensive gaps in our knowledge about what works to prevent suicide and how the different levels of intervention (USI) interact. However, there have been recent efforts to investigate the synergies between the different components of suicide prevention strategies (Harris et al., 2016). Given that suicide rates continue to rise in some countries (e.g., in the United States), perhaps it is time to reconsider whether a paradigm shift, rather than a ‘more of the same’ approach to suicide prevention is required.

As highlighted above, there has been welcome attention on indicated prevention strategies, focusing on those who are already suicidal. However, new thinking about universal and selective prevention is urgently required so that we can promote populations to be more resilient, to increase their coping skills and protect them from suicide risk. It is a major problem for those involved in suicide prevention including policy makers that there is so little evidence for universal and selective prevention strategies. Indeed, one of the few evidence-based strategies within universal prevention is the implementation of universal school awareness programs (Zalsman et al., 2016); much more research is urgently required.

More broadly, we also need to challenge attitudes, beliefs and knowledge regarding the preventability of suicide. Although suicide prevention is very difficult in practice, it is not impossible; but the existence of negative attitudes and beliefs that suicide is inevitable, that it cannot be prevented, are unhelpful. The establishment of the Zero Suicide (https://zerosuicide.sprc.org) movement has been very important in this regard, promoting the message that every suicide is one death too many. Related to the latter are the attitudes toward means restriction, especially in the context of restricting access to guns in the United States. Although restricting access to the means of suicide is one of the most effective suicide prevention strategies (Zalsman et al., 2016) in practice it is challenging to convince policy makers, gatekeepers, or mental health professionals of the need to implement a means restriction strategy. It is hoped that the recent focus on means safety (Anestis et al., 2018) rather than means restriction *per se* may help in this regard. The major challenge of integrating conversations about lethal means safety into standard primary care, mental health and substance use clinics was also identified by one of our experts.

There is also a lack of evidence regarding the most effective ways of implementing suicide prevention methods and strategies in different institutions/organizations including hospitals, mental health institutions, local governments and schools. However, there is evidence from the UK that implementing mental health service recommendations in particular the provision of 24 hour crisis care, having local policies for dual diagnosis and conducting a multidisciplinary review after a suicide are associated with falling suicide rates (While et al., 2012). Indeed, a major challenge is the reality that at least one quarter of people who die by suicide have had previous contact with mental health services (NCISH, 2017). We await with considerable interest the findings from on-going evaluations of the implementation of Zero Suicide (Mokkenstorm et al., 2017) initiatives as they will provide valuable insights into the challenges and facilitators of rolling out multi-level suicide prevention interventions in health care settings.

The chain of care and the need for continuity and high quality care are key to successful suicide prevention efforts especially among those who present to clinical services following self-harm. Indeed, in many countries throughout the world there are recommended standards of care and aftercare, but the reality is that these standards are frequently not implemented. Mehlum and Mork describe these challenges but crucially they have also identified key solutions which should improve adherence and sustainability over time (Mehlum and Mork, 2016). There is also considerable need for better linkages between crisis intervention, statutory services, therapists and the myriad of community and voluntary sector organizations that work in suicide prevention. Although it is difficult to set up studies to evaluate optimal chains of care, we urgently need more evidence about how to organize and implement linkages between different services and mental health settings.

There have been several studies regarding the clinical management of patients who present to hospital following suicidal behavior, nonetheless there is still a need for more evidence to inform best practice for the evaluation, treatment and follow-up of these patients. Although it is recommended that all patients who attempt suicide or self-harm should receive a comprehensive psychosocial assessment, at the hospital level there are wide variations in the clinical management such that the proportion of clinical presentations receiving psychosocial assessment (range 22–88%), medical (22–85%) or psychiatric (0–21%) admission and referral to non-statutory services (4–62%) (Cooper et al., 2013) varies markedly. However, there seems to be little association between these differences in hospital management and the repetition rate of self-harm (Cooper et al., 2015). As this is surprising, future studies should focus more on understanding the processes underlying the different management and treatment styles of suicidal people at hospital level and their relationship to patient outcomes.

As mentioned in the new developments in practice section the increased involvement of people with lived experience can only be positive for the field. Moreover, there is the possibility that including people with lived experience of suicidal ideation and/or behavior in suicide prevention programs might have other positive effects by providing a more powerful learning experience. Their involvement may yield similar effects to the Papageno effect that has been shown in relation to the media portrayal of suicide. The Papageno effect describes the positive and preventive effects of using positive testimonials of people with lived experience who have survived a suicide attempt and who have learned to overcome a suicidal crisis in a media portrayal of suicide. See Pirkis et al. (2016) for an overview of media influences and suicidal behavior literature.

Whereas the positive effect of including positive testimonials of people with lived experience in media portrayal is well established (Niederkrotenthaler et al., 2009, 2010) it ought to be extended to other areas of suicide prevention. Although there are some studies reporting the involvement of people with lived experience in the field of mental health these studies have tended not to evaluate this approach (Repper and Breeze, 2007). The Jones et al. (2018) study noted in the new developments in practice section is one of the few studies to explore the benefits of involving people with lived experience of suicidality in such studies. There is a strong need, therefore, for more evaluation studies investigating the effects, opportunities, and risks of involving people with lived experience in suicide prevention programs.

Given that male deaths by suicide vastly outnumber female suicides (Turecki and Brent, 2016), there is a huge challenge to gain more knowledge regarding how to approach and more effectively reach men with suicide prevention strategies. Men seek less help and communicate less about mental health problems so the challenge is how to motivate them for prevention and treatment. We also need to be careful that we do not blame men for not seeking help especially given that the support and services may not have been designed with them in mind. The stigma regarding mental health problems is also higher for men than for women, therefore, it is important that public health campaigns should direct their focus on men, specifically. As noted earlier, the extent to which existing interventions to reduce risk of suicide are effective for men is largely unknown.

Another challenge identified by our experts was the need to focus more on suicide risk in adolescents. In particular we need to identify and treat first onset suicidal ideation in adolescents. Indeed, if we are to better identify adolescents early on in the suicidal process we need to learn more about how to help them manage their suicidal ideation, how to enhance their coping skills, their social skills and capacity to solve social problems. We need to develop evidence-based interventions to increase their resilience that could help to buffer against suicide risk early in life. Targeting suicidal ideation in early adolescence should thus be a specific focus for suicide prevention efforts. As noted above, school-based suicide prevention programs should be considered in this regard (Katz et al., 2013; Robinson et al., 2013).

As a discipline, we should remain open to learning from other areas of public health and applying any such lessons to suicide prevention. For example, a very novel but intriguing suggestion by one of our experts is to study the effects of a dental health approach to determine whether it could be effective in improving mental health and prevent suicide. Translating the dental health approach into a mental health approach could involve us engaging in mental health self-care for a few minutes, say twice a day and this could be supplemented by an annual or 6 months check-up by a health care professional. Needless to say, a key challenge for such an approach would be around how we change existing attitudes, beliefs and knowledge regarding mental health to ensure that mental health self-care becomes an obvious and vital part of our daily health care, just like dental health care is. We should also look at what has been done with respect to stroke, heart disease and cancer; all areas of public health where the death rates have decreased markedly in recent decades while suicide rates have remained stable or increased.

Another fundamental methodological challenge for both practice and research is how to increase the integration of clinical knowledge into the clinical evidence-base. Indeed, well designed empirical studies and theoretical models to understand suicidal behavior often lack clinical knowledge; their translatability and implementation would benefit markedly from such knowledge. The complexity of the suicidal process and the individual differences associated with the development of suicidal ideation and behavior that are often observed in clinical practice are frequently overlooked in research, thereby limiting their clinical utility. A related issue is that there are few suicide-specific treatments that are actually available in the real world. It is incumbent on health care managers and policy makers to prioritize the accessibility of evidence-based treatments to those *who* need them *when* they need them. In short, the clinical management of patients who are suicidal remains a huge challenge, both in terms of the evidence base for tailored interventions and the accessibility of such interventions for those who are most vulnerable.

## Conclusion

It is an exciting time to be working in suicide research and prevention. In many countries throughout the world there have been important developments in understanding and preventing suicide. The four panels highlight key opportunities, challenges and pointers to move the field forward. We hope that their contents will guide the future research agenda, acting as a catalyst for new thinking in suicide prevention research and practice. However, it is important to reiterate the limitations of this Perspective article. Although the new developments and challenges outlined herein are extensive, they are not exhaustive but represent the views of 32 researchers or practitioners in the field. Nonetheless, the new developments identified by our experts are exciting, harnessing new technologies and approaches to better understand who is most at risk of suicide and why. However, many challenges remain; first, our ability to predict suicide is still not much better than chance and although there has been a welcome focus on suicide prevention interventions (both at the public health and clinical level), many gaps in our knowledge remain. None of us has all of the answers, and we hope that the suggestions reported herein will encourage new synergies and opportunities for interdisciplinary research. Finally, we are optimistic that the new developments and the field’s determination to overcome the identified challenges will combine to save more lives across the globe.

## Consent and Ethical Approval

All contributors named in the section “Acknowledgments” were asked for their views on new developments and challenges in suicide research and prevention. They all provided written informed consent (by email) that their views could be collated by the authors for use in publication. Consistent with the University of Glasgow’s Medical, Veterinary and Life Sciences ethics committee’s institutional policy, no ethical approval was sought as contributors were giving their views in their expert capacities. It is important to note, however, that the views and interpretations expressed in the article are those of the authors and not the contributors.

## Author Contributions

Both authors listed have made a substantial, direct and intellectual contribution to the work, and approved it for publication.

## Conflict of Interest Statement

The authors declare that the research was conducted in the absence of any commercial or financial relationships that could be construed as a potential conflict of interest.

## Footnotes

1 Some authors also noted new developments and challenges in policy that are beyond the scope of this article.
